# Essentials of Writing a Successful Doctoral Thesis

**DOI:** 10.7759/cureus.89626

**Published:** 2025-08-08

**Authors:** Iqbal Ratnani, Sahar Fatima, Emman Fatima, Ian Hill, Zehra Surani

**Affiliations:** 1 Clinical Anesthesiology and Critical Care, Houston Methodist Hospital, Houston, USA; 2 Critical Care, Houston Methodist Hospital, Houston, USA; 3 Biomedical Sciences, Creighton University, Omaha, USA; 4 Anesthesia, Creighton University School of Medicine, Omaha, USA; 5 Medical Education and Simulation, It's Your Life Foundation, Corpus Christi, USA

**Keywords:** academic writing, co-guide for thesis work, research writing, thesis guide, writing and literature

## Abstract

As more professionals acquire mid-career extra degrees, pursuing a doctorate in the given field is becoming the norm. One of the major challenges of obtaining a mid-career degree is balancing family and professional life, along with the added responsibility of fulfilling curricular educational requirements for a degree. This article aims to highlight and overcome those challenges. Undertaking this major commitment requires discipline, planning, and, by all means, changing one's lifestyle. Lifestyle changes specifically include becoming more organized and balancing work and study, along with financial and mental health considerations. Keeping sanity in all this "organized chaos" requires developing inner strength and constant dialogue with oneself. The authors used their own experiences to draft a framework that can serve as a guide to help those in pursuit of higher education. The flow diagram has been aimed at concisely putting the stepwise development of the thesis, with the hope that it helps other mid-career students and professionals.

## Editorial

A successful thesis requires thorough research, a good literature review, and effective communication of one’s work through writing [1]. Though most theses are intended for a specific audience, a good doctoral work should be comprehensible to the average reader while maintaining its intellectual strengths. An ideal doctoral thesis combines a unique idea, smooth transitions from one area to another, without grammatical and spelling errors, and well-researched, evidence-based arguments. Ensuring an honest report of the limitations of the findings is also an integral part of a good thesis. 

“Writing is an art,” as E. E. Cummings once stated. The Merriam-Webster dictionary defines art as “the conscious use of skill and creative imagination, especially in the production of aesthetic objects” [2]. This skill becomes highly relevant when summarizing research in a doctoral thesis, as it provides insight into the quality of an individual’s original research. It also allows one to clearly and effectively communicate complex ideas, theories, and research findings to both academic and non-academic audiences. 

After the research question has been decided, tactfully organizing the outline is one of the most fundamental steps in writing a thesis. An outline helps ensure that the final purpose of the paper is met. The paper should begin with an abstract that is brief and free of unnecessary verbiage. The abstract serves as the “tip of the iceberg” for the reader to understand the paper's content. Some experts recommend writing abstracts after completing the thesis. This is because it serves as a concise summary of the paper’s overview. After the abstract, the focus should be on writing a clear-cut introduction, as it paints a crucial picture: setting the stage for the rest of the paper [3]. Providing a concise topic background before posing the research question is extremely important and should fulfill the basic parameters: What’s new? So what? Why so? Why now? And who cares? [4].

The main body of the research should have the following three qualities: relevant references from the literature, evidence of the author’s dedication to the thesis apart from desk work (e.g., personal expeditions to required places and visits that could be virtual) to authoritative figures in the field), and tables, flowcharts, and pictorials for better comprehension [5]. Each section should be detailed enough to allow replication of the study and provide a clear understanding of how the research was conducted. Data should be analyzed using an appropriate statistical test. Using the wrong test can skew the results of the thesis. If statistical results are included, they should be checked twice, preferably by a trained statistician. Their expertise can help identify any mistakes or misinterpretations and provide validation of the statistical methods used. By rigorously checking your statistical results and deeply exploring the implications of your research question, you enhance the credibility and impact of your thesis [6-8].

The implications of the research question should be explored with logical rigor and depth, aiming to uncover novel insights that have not been previously articulated. A thesis should end with a short conclusion, leaving the audience wanting to explore the topic further. Although the bibliography is often positioned at the end and may be overlooked by many readers, it embodies the author's commitment and effort in researching the topic. It acknowledges the contributions of other experts in the field. Proper vetting to obtain reliable references is of paramount importance. These references should be compiled accurately, and a specific citation style should be followed to ensure that guidelines are met. Since readers or members of the reviewing committee may not be familiar with specialized terminology used in the work, these terms should be clearly defined at the outset. Consider including a glossary at the end of the document for particularly complex or lengthy terms. Additionally, abbreviations should be spelled out in full when they first appear in the text [9,10].

Review

In a doctoral thesis, it is crucial to use hedges to avoid giving the impression of presenting definitive conclusions. Conversely, boosters can be employed to emphasize the strength of the references and sources used in the literature. Sentences should be shortened using single verbs; if needed, connectors can be used to avoid disrupting the flow. Excessive adverbs can ruin any good thesis and should be used judiciously. Using the passive voice can be tricky and should be used cautiously in any given sentence. 

In the end, a good thesis should flow like a wave, so that readers feel they are discovering new information with each successive sentence. While scholarly, the thesis should also engage the reader and maintain their interest. It should be compelling and well-argued, making a clear case for the significance of the research. This requires regularly revisiting the draft to refine and eliminate redundant or cluttered information. The final presentation of a doctoral thesis should be approached with an artistic sensibility.

Some basic skills and experiences are required to create an impressive doctoral thesis. Basic skills include grasping scientific writing, proper grammar, and an understanding of general scientific terms. If these skills are not present, formal help through a dedicated program should be sought before embarking on the writing of a doctoral dissertation. Previous experience refers to reading academic journal articles and formally participating in or being a part of an academic environment. It does not imply that a candidate is solely focused on writing or analyzing academic texts; rather, the influence of academic literature on the candidate's intellectual development is important. Experience such as mentoring students, assisting with writing tasks like abstracts and articles, or participating in review activities can significantly improve scholarly skills. Additionally, many academic institutions offer certification programs lasting several months or even a year to help develop scholarly research skills. Enrolling in such programs can facilitate a more successful thesis defense at various stages. In addition, practical experience with academic research and publication, such as contributing to collaborative projects or presenting at conferences, further enhances one's ability to conduct and present research effectively. Many academic institutions offer certification programs, workshops, and specialized courses designed to develop scholarly research acumen over several months or a year. Participating in these programs can provide valuable skills and knowledge, ultimately supporting a more successful thesis at various stages and contributing to overall academic and professional growth. Any experience in these courses to create a capstone project quickly makes a candidate well-versed and enhances the basic skills that are crucial in creating an academic thesis. Above all, practicing one’s writing skills and engaging in writing exercises is paramount to improving individual skill and ensuring a higher-quality academic paper. Digital media, additionally, can be used to practice academic writing through blogs, letters to editors, commenting on articles, creating multiple-choice questions/answers/explanations (MCQs), and even drafting a paragraph for an academic short video. These digital platforms allow for real-time collaboration and communication with peers or mentors to improve academic writing skills [11-14]. Having a mentor for guidance plays a crucial role. Mentors can help develop essential writing skills, such as critical analysis and argumentation. Additionally, they can share their own experiences and tactics for overcoming challenges when writing a thesis. All these endeavors work together and play strong roles in providing a core foundation for the creation of any doctoral work.

In this regard, taking the self-assessment exercise (SAE) through the academic writing center of the institution where the candidate is pursuing a doctorate, or using any online rubric, is highly recommended to assess and understand one’s strengths and weaknesses comprehensively (one such example is the Texas A&M University self-assessment kit) [15]. It is advisable to take such SAE before embarking upon the journey of creating an academic thesis. Such self-assessment tests a candidate in various components of learning style and finding information. Essential elements in this regard include answering important questions, such as developing a plan for studying independently, locating relevant research materials, and maintaining a bibliographic record.

It becomes particularly engaging for candidates when they critically evaluate their thinking skills, including their ability to maintain clarity of purpose for any given task, ask relevant questions, read critically, remain aware of biases, and distinguish between facts and opinions. The SAE should also address reading skills, for example, how quickly a candidate can read specific details or catch the messages “between the lines,” and if a candidate is good at guessing the meaning of unfamiliar words or take practical notes. Often overlooked, SAE should also assess listening skills, such as maintaining concentration while listening to lectures and reflecting on the topic beforehand. Additionally, the SAE should assess candidate discussion skills, including oral presentation and writing skills, as an essential task to become a good defender of an effectively written thesis. SAE effectively led candidates to recognize weaknesses, including a lack of skill in creating aesthetically pleasing visual aids, ineffective speaking, writing ineffective introductions and/or conclusions, and poor punctuation and grammar. Many candidates are unfamiliar with the various citation formatting styles.

Assessing one’s weaknesses in writing academic manuscripts is the first step toward preparing oneself for an impressive doctoral thesis. One must sharpen their reading and writing skills to the optimum. A candidate must be ready to adapt their current style, flow, and presentation methods. It is not uncommon for someone working to improve academic writing to pay close attention to flow when reviewing emails, texts, articles, or books. They will scrutinize the use of boosters or hedges, tense consistency, voice, and sentence connectors to refine their writing. Two books that may be recommended to be handy while taking this journey are The Sense of Style by Steven Pinker and the Publication Manual of the American Psychological Association. Mentors, teachers, and candidate committee members [16,17] all play a crucial role in guiding an individual through the thesis writing process methodically. A candidate should highly depend on their feedback and constructive criticisms. It is important to respect mentors’ time and utilize virtual meeting rooms or digital communication if they can’t meet personally.

An often-underutilized resource is the writing center available at most institutions, which should be used more regularly. Additionally, exchanging various writings with classmates or colleagues can provide valuable feedback on content and style. If necessary, consider enrolling in additional formal classes to improve visual aids, refine grammar, and enhance speaking skills, which are crucial for effectively defending a thesis. As a candidate researches their doctoral thesis, they should learn how to be more organized in the digital world by creating folders, taking notes, and preserving them properly in a way that is easily accessible for rereading, revising, and editing at any time. However, it may require some effort to visit the library (or access it digitally) to review doctoral theses that have already been successfully defended. If possible, contact the authors of these doctoral works to gain their insight and suggestions to better understand the process. Additionally, attending various thesis defenses can provide insights into strategies and techniques used by other candidates during their presentations. In short, over several months, a doctoral thesis may become a lifestyle changer, requiring immense discipline, focus, organization, and the development of various writing, reading, and communication skills [18,19].

**Figure 1:**
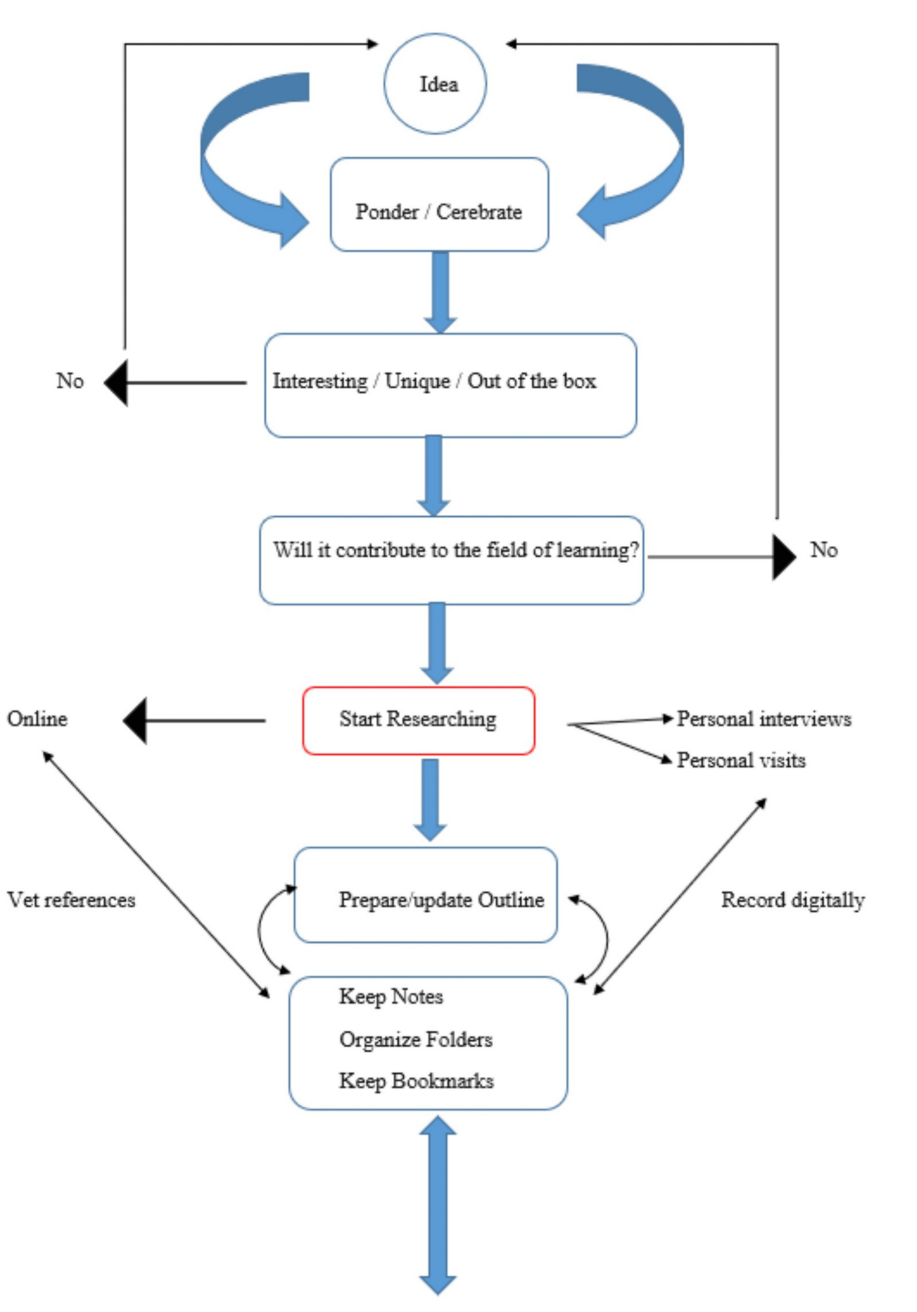
First stage of thesis development Original figure designed by author

**Figure 2:**
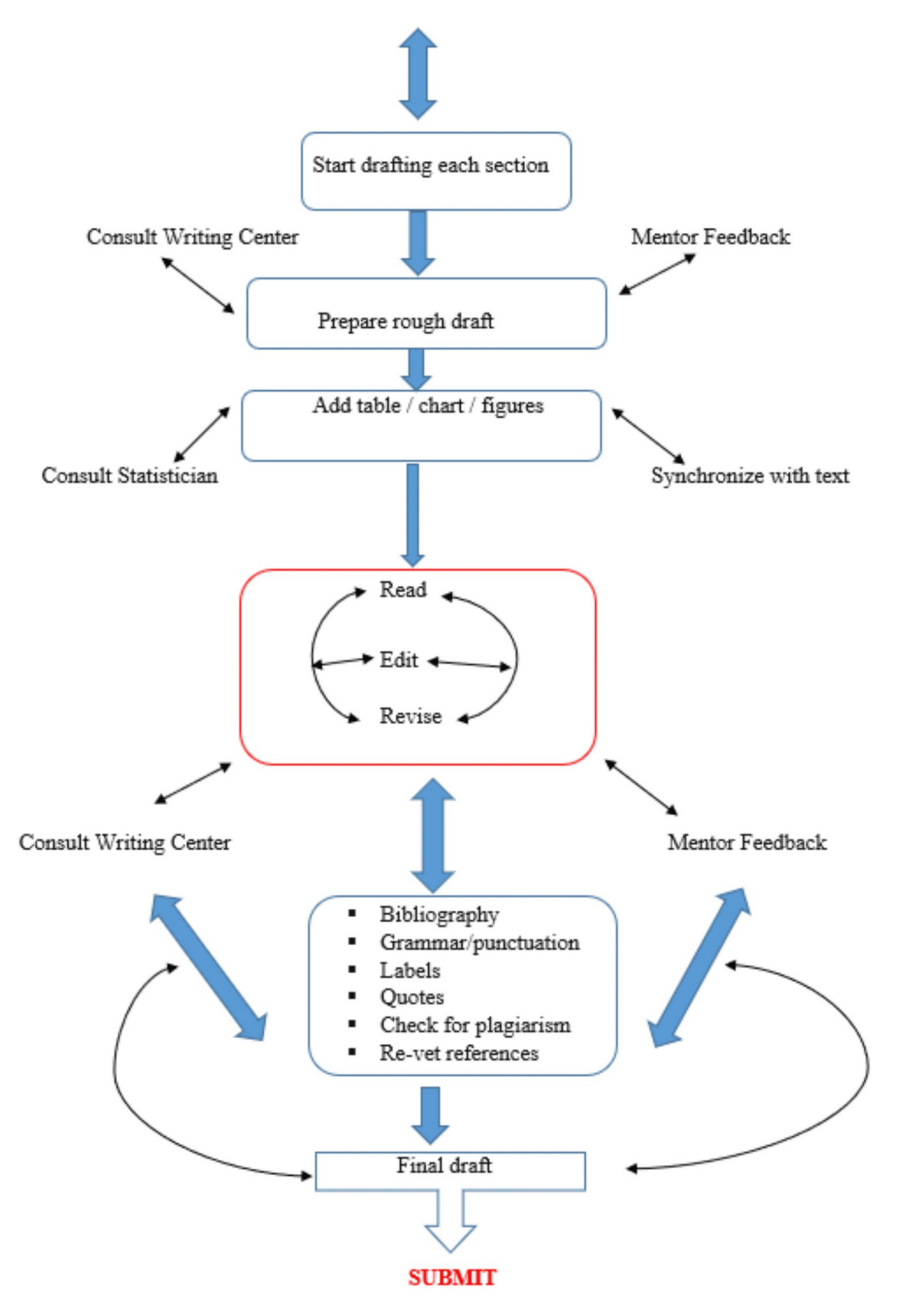
Second stage of thesis development Original figure designed by author

Thesis development can be summarized by the flow diagrams below (Figures 1-2). It can take place in two stages, with one stage leading to the other. However, it is important to note that the two-way arrow connecting the stages signifies that the sequence of steps within the stages can be reversed (Figures 1-2).

Conclusions

At the end of the day, what matters is the level of priority a thesis holds in a candidate’s life while they carry out their day-to-day matters. Eventually, a candidate needs to learn time management and proper planning, including scheduling adequate hours of sleep. Creating a calendar to manage time and plan days effectively is a valuable tool to consider. Adding another highly inspiring goal can be extremely challenging alongside full-time work, family, and other responsibilities. Prioritizing different obligations is important. A candidate may find themselves preoccupied with their thesis during various activities, such as driving, exercising, traveling, or even taking a coffee break at work. Continuous reflection on the thesis, jotting down thoughts to avoid forgetting them, and maintaining a constant mental image of the work in progress become essential habits. It may be wise and prudent to warn and prepare loved ones and colleagues at work for this incoming obsession, which may be called “thesis-mania.” A good sense of humor during difficult times can be a lifesaver!
